# Altered resting‐state functional connectivity and effective connectivity of the habenula in irritable bowel syndrome: A cross‐sectional and machine learning study

**DOI:** 10.1002/hbm.25038

**Published:** 2020-06-03

**Authors:** Cui P. Mao, Fen R. Chen, Jiao H. Huo, Liang Zhang, Gui R. Zhang, Bing Zhang, Xiao Q. Zhou

**Affiliations:** ^1^ Department of Medical Imaging Second Affiliated Hospital of Xi'an Jiaotong University Xi'an Shaanxi China; ^2^ Department of Gastroenterology Second Affiliated Hospital of Xi'an Jiaotong University Xi'an Shaanxi China; ^3^ School of Computer Science and Engineering Xidian University Xi'an Shaanxi China

**Keywords:** effective connectivity, habenula, irritable bowel syndrome, machine learning, resting‐state functional connectivity

## Abstract

Irritable bowel syndrome (IBS) is a disorder involving dysfunctional brain–gut interactions characterized by chronic recurrent abdominal pain, altered bowel habits, and negative emotion. Previous studies have linked the habenula to the pathophysiology of negative emotion and pain. However, no studies to date have investigated habenular function in IBS patients. In this study, we investigated the resting‐state functional connectivity (rsFC) and effective connectivity of the habenula in 34 subjects with IBS and 34 healthy controls and assessed the feasibility of differentiating IBS patients from healthy controls using a machine learning method. Our results showed significantly enhanced rsFC of the habenula‐left dorsolateral prefrontal cortex (dlPFC) and habenula‐periaqueductal grey (PAG, dorsomedial part), as well as decreased rsFC of the habenula‐right thalamus (dorsolateral part), in the IBS patients compared with the healthy controls. Habenula‐thalamus rsFC was positively correlated with pain intensity (*r* = .467, *p* = .005). Dynamic causal modeling (DCM) revealed significantly decreased effective connectivity from the right habenula to the right thalamus in the IBS patients compared to the healthy controls that was negatively correlated with disease duration (*r* = −.407, *p* = .017). In addition, IBS was classified with an accuracy of 71.5% based on the rsFC of the habenula‐dlPFC, habenula‐thalamus, and habenula‐PAG in a support vector machine (SVM), which was further validated in an independent cohort of subjects (*N* = 44, accuracy = 65.2%, *p* = .026). Taken together, these findings establish altered habenular rsFC and effective connectivity in IBS, which extends our mechanistic understanding of the habenula's role in IBS.

## Graphical abstract

We investigated the resting‐state functional connectivity (rsFC) and effective connectivity of the habenula in 34 subjects with Irritable Bowel Syndrome (IBS) and 34 healthy controls and assessed the feasibility of differentiating IBS patients from healthy controls using a machine learning method. Our findings suggested altered habenular resting‐state functional connectivity and effective connectivity in IBS, which extends our mechanistic understanding of the habenula's role in IBS.

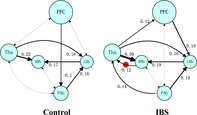

## 1 INTRODUCTION

Irritable bowel syndrome (IBS) is one of the most widely diagnosed disorders that involves dysfunction in brain–gut interactions and is characterized by chronic recurrent abdominal pain, altered bowel habits, and negative emotion (Fond et al., 2014; Fukudo & Kanazawa, 2011; Mearin et al., 2016). The heterogeneity of IBS and poor understanding of its pathophysiology make treatment choices difficult. Recently, neuroimaging research in gastroenterology has uncovered mechanisms through the neural activity and circuitry associated with IBS by the presence of structural and functional alterations in multiple brain regions, including the anterior cingulate cortex, amygdala, insula, prefrontal cortex, hippocampus, and so forth (Fukudo & Kanazawa, 2011; Labus et al., 2014; Mayer et al., 2019; Qi, Liu, Ke, Xu, Ye, et al., 2016; Qi, Liu, Ke, Xu, Zhong, et al., 2016).

The habenula, a small epithalamic structure, has been implicated in the pathophysiology of negative emotion and pain by accumulating evidence (Fakhoury, 2017; Namboodiri, Rodriguez‐Romaguera, & Stuber, 2016). The majority of previous studies relating to the habenula have been carried out in the context of psychiatric disorders, including major depression (Lawson et al., 2017; Liu, Valton, Wang, Zhu, & Roiser, 2017), addiction (McLaughlin, Dani, & De Biasi, 2017), schizophrenia (Li et al., 2019; Schafer et al., 2018; Zhang et al., 2017) and other mood disorders (Ambrosi et al., 2019), while only a small number of investigations have begun to focus on its role in pain and analgesia (Cohen & Melzack, 1993; Khalilzadeh & Saiah, 2017; Shelton, Pendse, et al., 2012; Shelton, Becerra, & Borsook, 2012). For instance, acute experimental pain can activate the bilateral habenula in humans (Shelton, Pendse, et al., 2012), and analgesic effects can be generated by microinjecting morphine into the habenula (Khalilzadeh & Saiah, 2017) or by suppressing the M‐channels in the habenula in rats (Kang et al., 2019). Despite the significant progress of research in animals, habenula studies in humans are still nascent. As a critical neuroanatomical hub involved in negative emotion and pain, habenular function in IBS remains to be established.

To date, resting‐state functional magnetic resonance imaging (rsfMRI) (Fox & Greicius, 2010) and machine learning methods have been used to study brain signatures in chronic pain states (Lotsch & Ultsch, 2018; Zhang et al., 2019). rsfMRI makes it possible to study the brain's resting‐state functional connectivity (rsFC), while machine learning can automatically detect a number of neurological diseases based on structural and functional neuroimaging to better understand the complexity of pain. Of the various methods in machine learning that have been used in pain research (Lotsch & Ultsch, 2018), the support vector machine (SVM) classifier (Chang & Lin, 2011) has been most commonly used to explore brain regions in discriminating patients with chronic low back pain and healthy controls (Shen et al., 2019; Ung et al., 2014; Zhang et al., 2019). Altered habenular rsFC has been reported in subjects with chronic pain, such as complex regional pain syndrome (CRPS) (Erpelding et al., 2014), and several psychiatric disorders (Ely et al., 2016; Zhang et al., 2017). RsfMRI and machine learning were used to explore the habenular connectivity patterns in this study.

However, conventional connectivity analyses cannot address the causal influence of one neural system on another. Dynamic causal modeling (DCM) can characterize the causal relationships between brain regions on the basis of fMRI data and has become a predominant way of quantifying effective connectivity (Friston, Harrison, & Penny, 2003). In this study, we hypothesized that there might be altered connectivity of the habenula in patients with IBS and investigated (a) the rsFC and effective connectivity of the habenula in subjects with IBS; (b) the feasibility of classifying IBS subjects and healthy controls by machine learning methods; and (c) the relationship between the habenular connectivity and clinical measures in IBS patients.

## 2 MATERIALS AND METHODS

### 2.1 Subjects

Thirty‐four patients with IBS (17 females; age: 44.9 ± 13.2 years, mean ± *SD*) and 34 demographically similar healthy controls (17 females; age: 44.9 ± 14.3 years, mean ± *SD*) were included in this study. The data presented here are part of an ongoing study, and a portion of the data were used in our previous publication (Mao et al., in press). In brief, we recruited participants with IBS from the Outpatient Clinic of Gastroenterology in the Second Affiliated Hospital of Xi'an Jiaotong University and healthy control subjects from the surrounding area through advertisements and word‐of‐mouth. The study was approved by the Research Ethics Committee of Xi'an Jiaotong University (No. 2015049). The ROME IV criteria (Drossman & Hasler, 2016) were used to diagnose IBS. Patients with IBS with all types of bowel habits (diarrhoea, constipation, mixed constipation and diarrhoea) were included. Subjects were excluded if they (a) had contraindications to perform MR imaging; (b) had a history of gastrointestinal surgery, psychiatric disorders, substance abuse, treatment with antidepressants or other medications; (c) had other organic diseases; or (d) had chronic pain in other body sites. All participants gave their written informed consent. The medications of all patients with IBS were recorded and can be seen in the Supplemental Material.

### 2.2 Questionnaires

All subjects reported his or her most bothersome symptoms and bowel habits. Abdominal pain in the patients with IBS was evaluated by the short form of the McGill Pain Questionnaire (SF‐MPQ) (Melzack, 1987). The subjects rated the intensity of pain on a visual analogue scale (VAS) on the day of the scan. The depressive state of the patients with IBS was evaluated by the Hamilton Depression Rating Scale (HDRS) (Williams, 1988).

### 2.3 MRI

All participants underwent MR scans on a 3T MR scanner (Signa HDXT, GE Heathcare) equipped with an 8‐channel head coil. MR scans were performed when the IBS patients were in their non‐episodic period. All IBS patients were required not to take medications for 12 hr before the MR scan. High‐resolution T1‐weighted images were obtained (time to echo/time to repetition [TE/TR] = 4.8/10.8 ms; slice gap = 1 mm; voxel resolution = 1 mm^3^). The resting‐state functional MR data were acquired by gradient echo‐planar imaging (repetition time, 2,500 ms; echo time, 30 ms; slice thickness, 3 mm; flip angle, 90°; matrix, 64 × 64; voxel size, 4 × 4 × 3 mm^3^; 50 slices; acquisition time = 6 min 35 s).

### 2.4 Data processing

The functional MR data were processed using the CONN toolbox v18b (Whitfield‐Gabrieli & Nieto‐Castanon, 2012) and DCM in SPM12 software (Wellcome Department of Imaging Neuroscience, London, UK; http://www.fil.ion.ucl.ac.uk/spm/). The first 4 time points were discarded due to transient signal changes before magnetization reached a steady‐state. The preprocessing included the realignment and unwarping of the functional images, slice‐timing correction, motion correction, co‐registration with the structural data (target resolution for functional images = 1 mm), spatial normalization, and smoothing (full‐width‐at‐half maximum [FWHM] = 4 mm). The default CONN preprocessing steps were set up to automatically use a combination of aComCor (white matter and cerebral spinal fluid regions of interest (ROIs), 5 components each), scrubbing, motion regression (12 regressors: 6 motion parameters + 6 first‐order temporal derivatives), and filtering to reduce the noise (band: 0.008–0.09 Hz). The regressors used in the scrubbing regression were the “outliers”, that is, the images if composite movement from a preceding image exceeded 0.5 mm, or if the global mean intensity was >3 standard deviations from the mean image intensity for the entire resting scan (Liu et al., 2019; Wang et al., 2017). Considering the small size of the habenula, the target template resolution for functional images was resampled to 1 × 1 × 1 mm^3^. The following first‐level and second‐level analyses were carried out based on these data. To reduce the effect of motion artefacts in IBS patients and healthy controls, we also calculated the mean frame‐wise displacement (FD) based on Jenkinson, Bannister, Brady, and Smith (2002) and included it as a covariate in all group‐level analyses.

**FIGURE 1:**
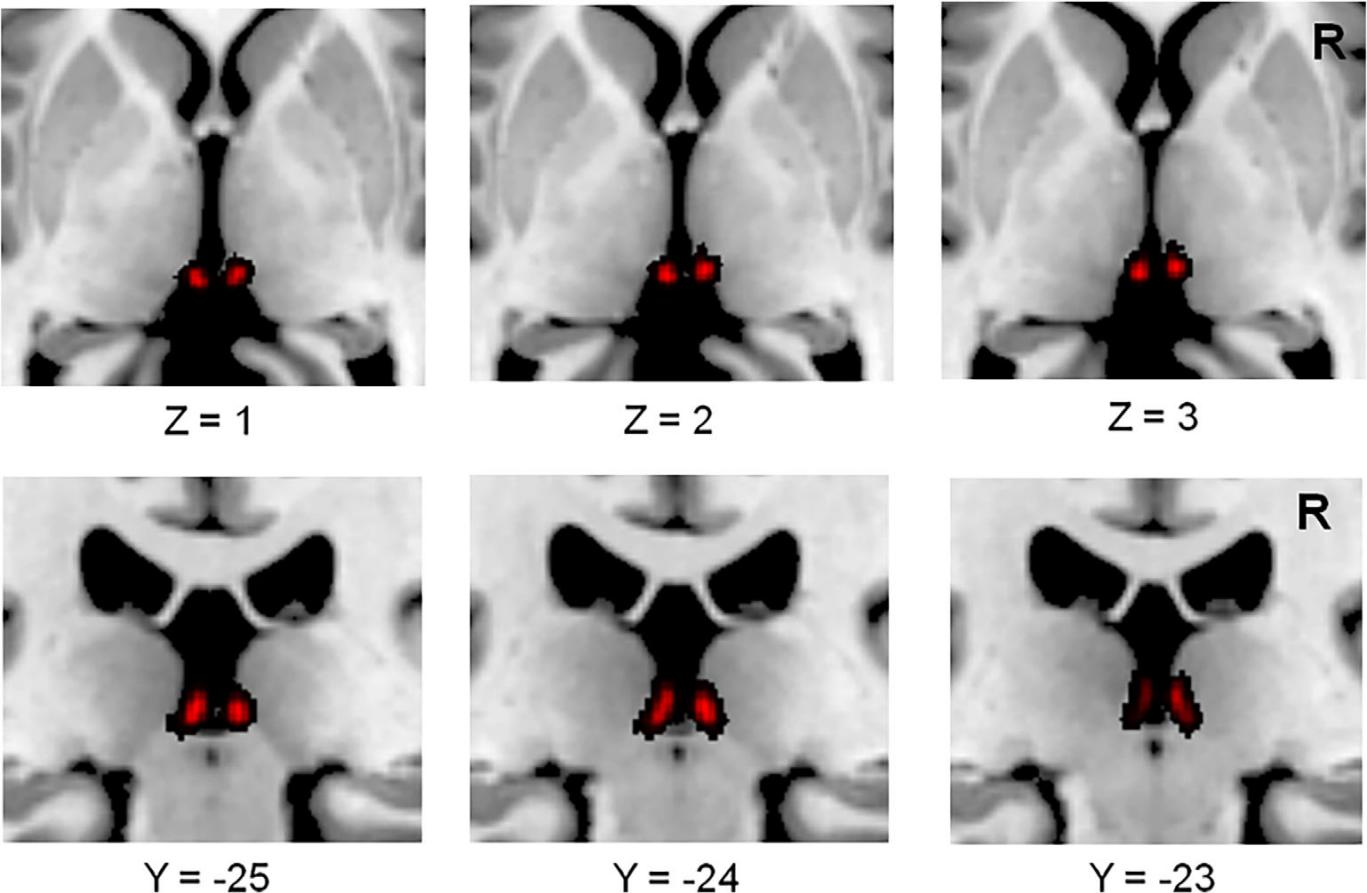
The habenular regions of interest. Anatomical regions of interest were used for the left and right habenula (red), and then they were combined into one seed. R, right

To improve the power to detect connectivity given the small size of the habenula, the left and right habenula were combined to create a single habenula seed. Similar methods can be found in previous studies (Ely et al., 2016; Torrisi et al., 2017). The seeds for the left and right habenula proposed by Kim et al. (2016) were used in this study (Figure 1), which were thought to be suitable for both morphological evaluation and habenula seed region selection in functional and diffusion MRI applications. For each individual subject, a seed‐to‐voxel functional connectivity analysis was carried out to compute the correlation maps between the seed and voxels in the rest of the brain. After that, *z*‐transformed connectivity maps were obtained and compared between groups by two‐sample *t* test, with age, gender and depression scores as covariates. The results were considered significant at a threshold of voxel‐wise *p* < .001 uncorrected and cluster‐level *p* < .05, false discovery rate (FDR) corrected for between‐group comparisons.

Since the thalamus and periaqueductal grey (PAG) are repeatedly reported to have functional connectivity with the habenula (Shelton, Pendse, et al., 2012; Torrisi et al., 2017) and to be crucial in pain modulation (Gustin et al., 2011; Samineni, Premkumar, & Faingold, 2017), we predefined these two regions as ROIs. The left and right thalamus were defined based on the automated anatomical labeling and IBASPM 71 brain atlas (Collins, Holmes, Peters, & Evans, 1995) and were combined into one seed mask. The seed of the PAG was defined according to the parcellation protocol described by Keuken et al. (2017). For these two ROIs, their connectivities with the habenula were explored by a small‐volume correction analysis implemented in SPM, with a significance level of cluster‐wise *p* < .05 after FDR correction in the group comparisons.

### 2.5 Dynamic causal modeling

The spectral DCM analyses were performed using DCM 12 (Friston, Kahan, Biswal, & Razi, 2014) implemented in SPM12. According to the between‐group differences in habenular connectivity, we selected five areas as ROIs, including the left dorsolateral prefrontal cortex (dlPFC), right thalamus, left habenula, right habenula, and PAG. Time series were extracted from the anatomical ROIs of the left and right habenula, and clusters of the dlPFC, PAG and thalamus were obtained from the CONN analyses. The data used to extract time series were unsmoothed but otherwise fully preprocessed fMRI data. The following seven models were specified: a fully connected model, three models including the bilateral habenula and one of the other three regions (dlPFC, thalamus, PAG), and three models including the bilateral habenula and any two of the other three regions (dlPFC, thalamus, PAG). Fixed effects (FFX) Bayesian Model Selection (BMS) was conducted to determine the best model for each subject. For the best model, Bayesian Model Averaging (BMA) (Penny et al., 2010) was conducted to analyze the connectivity parameters. The probability‐weighted values of the model parameters were also obtained from BMA. We only report effects that have a posterior probability > .95.

### 2.6 Comparisons of habenular rsFC and effective connectivity in an independent cohort of IBS patients and healthy controls

To validate the results from the CONN and DCM analyses, we repeated these two analyses in an independent cohort that included 22 patients with IBS and 22 healthy controls.

### 2.7 Machine learning

SVM was used in this study to classify IBS and healthy controls in MATLAB based on a library (LIBSVM) (Chang & Lin, 2011). The brain regions showing significant group differences (2‐mm radius sphere) were selected as ROIs and the habenular rsFCs were extracted and used in the classification by SVM. The data set in our manuscript was randomly split into training set and testing set for 10 times. The number of cases in the training and testing sets were consistent, respectively (68 cases for training with 34 cases of control group and 34 cases of IBS, 44 cases for testing with 22 cases of control group and 22 cases of IBS). In order to prevent over fitting, 10‐fold cross‐validation (Pereira, Mitchell, & Botvinick, 2009) was applied on the training set. In order to quantify the performance of the final machine learning model, the accuracy, sensitivity, specificity, and area under the curve (AUC) were calculated to reduce the impact of deviations in the distribution of the training and testing sets. In addition, the accuracy (ACC) of testing set was assessed by permutation test with 1,000 epochs as described in previous studies (Golland & Fischl, 2003).

We have trained the machine learning model for each feature (connectivity values of the habenula with dlPFC, thalamus, and PAG) independently. And then, in order to see whether we can get a better performance with all these three features, we trained the model again by combing the connectivity values of the habenula with these three regions.

### 2.8 Statistical analysis of demographic and clinical data

Statistical analyses not included in the CONN tools and DCM were performed with SPSS 13.0 (SPSS Inc., Chicago, IL). Independent sample *t* tests or Mann–Whitney *U* tests were used to evaluate the differences in age and HDRS scores according to the normality and homogeneity of variances evaluated by Kolmogorov–Smirnov and Levene's tests, respectively. Gender differences between the two groups were assessed by the chi‐squared test. *p*‐values < .05 were considered statistically significant. We extracted the rsFC between the habenula and each region demonstrating a significant between‐group difference in the resting‐state analyses with a 2‐mm (radius) sphere and performed correlation analysis between them and disease characteristics (VAS scores and disease duration). The significant effective connectivity was also correlated with clinical measures in the IBS group. The *p* values from correlation analysis were adjusted by FDR correction.

## 3 RESULTS

### 3.1 Demographic and clinical data

No significant group differences were found for age (*p* = .986, 2‐sample *t* test), gender (*p* = .596, *χ* ^2^ test), or HDRS scores (*p* = .09, 2‐sample *t* test). The VAS and SF‐MPQ scores were 3.7 ± 2.0 and 8.4 ± 5.1 (mean ± *SD*), respectively, in the IBS patients. The disease duration was 6.0 ± 7.3 years (mean ± *SD*) in the IBS group. The detailed information can be seen in Table S1.

### 3.2 Resting‐state functional connectivity of the habenula

**FIGURE 2:**
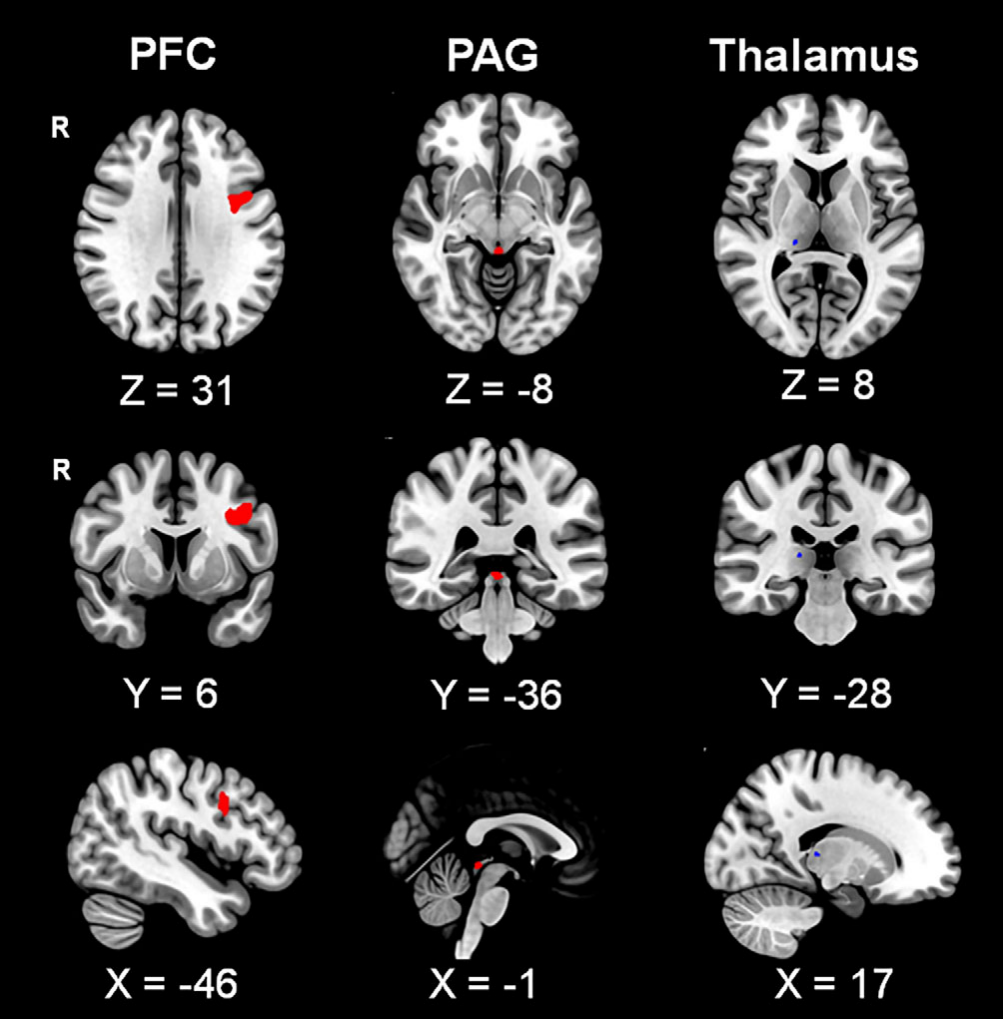
Resting‐state functional connectivity of the habenula. Both enhanced (red) and decreased (blue) resting‐state functional connectivity (rsFC) of the habenula were found in patients with irritable bowel syndrome (IBS) when compared to healthy controls. The connectivity values of the habenula with the periaqueductal grey and right thalamus were obtained from small‐volume correction analyses. PFC, prefrontal cortex; PAG, periaqueductal grey; R, right

There were no significant differences in the mean FD (*p* = .73) between the patients with IBS (0.073 ± 0.055 mm) and healthy controls (0.077 ± 0.041 mm). Relative to the healthy controls, the patients with IBS showed significantly enhanced functional connectivity of the habenula‐left dlPFC (*p* = .005, FDR corrected; Figure 2; Table 1) and habenula‐dorsomedial PAG (*p* = .003, FDR corrected). There was decreased rsFC of the habenula‐right thalamus (dorsolateral part) in the patients with IBS compared to the healthy controls (*p* = .014, FDR corrected; Figure 2; Table 1).

**TABLE 1:** Group differences in the resting‐state functional connectivity of the habenula

Group difference	Target area	Volume data			Voxels	*Z* value	*p*
		*X*	*Y*	*Z*			
Control < IBS	Left dlPFC	−36	1	32	1,427	3.9	.005
	PAG	−1	−30	−7	109	4.09	.003
Control > IBS	Right thalamus	17	−28	8	25	2.7	.014

*Note:* The *p* values are corrected for false discovery rate.

Abbreviations: dlPFC, dorsolateral prefrontal cortex; IBS, irritable bowel syndrome; PAG, periaqueductal grey.

### 3.3 Effective connectivity of the habenula

**FIGURE 3:**
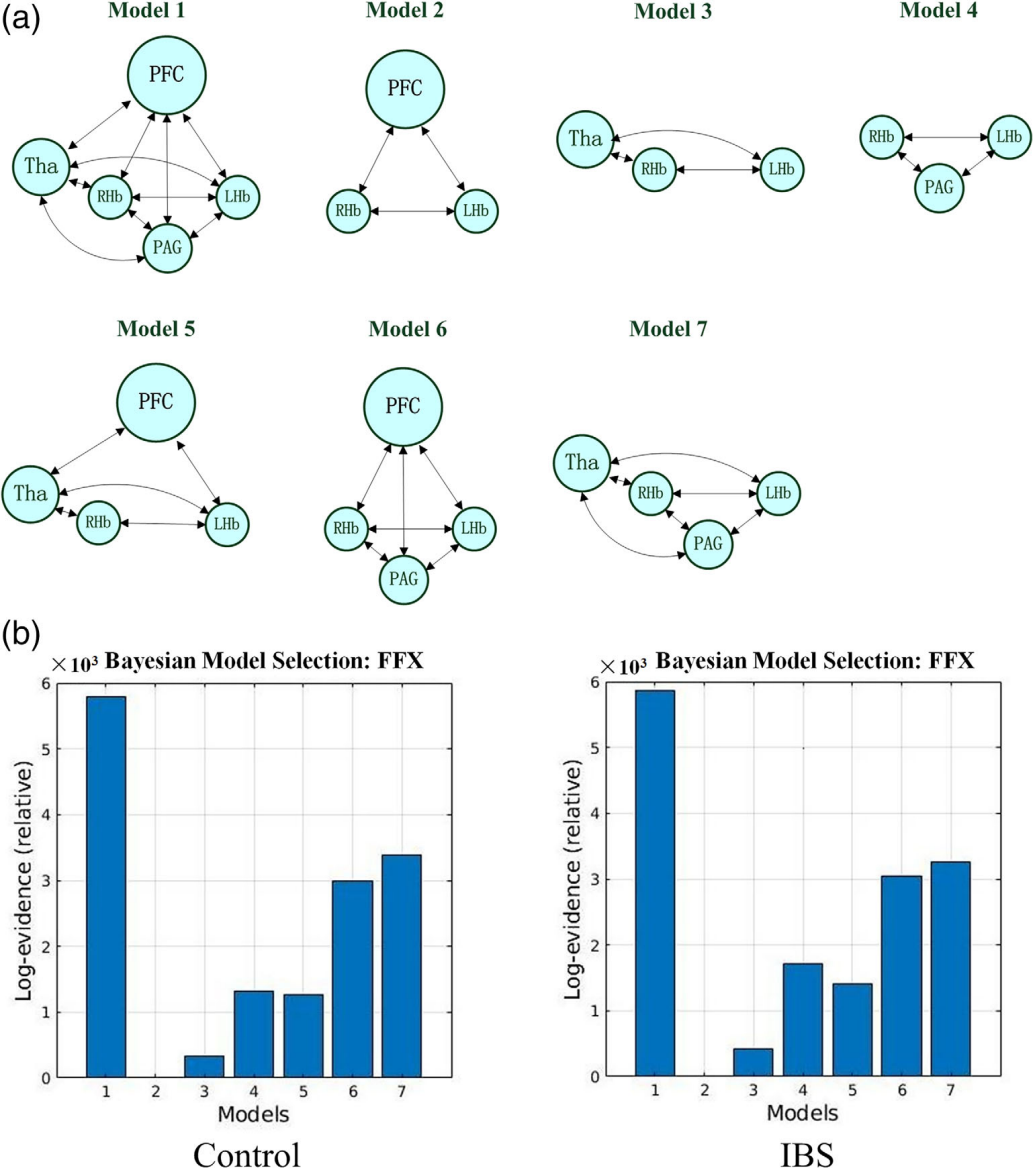
The investigated models in DCM. (a) Seven models were investigated in this study. (b) The results revealed by Bayesian Model Selection at the single‐group level. IBS, irritable bowel syndrome; PFC, prefrontal cortex; Tha, thalamus; LHb, left habenula; RHb, right habenula; PAG, periaqueductal grey; FFX, fixed effects

**FIGURE 4:**
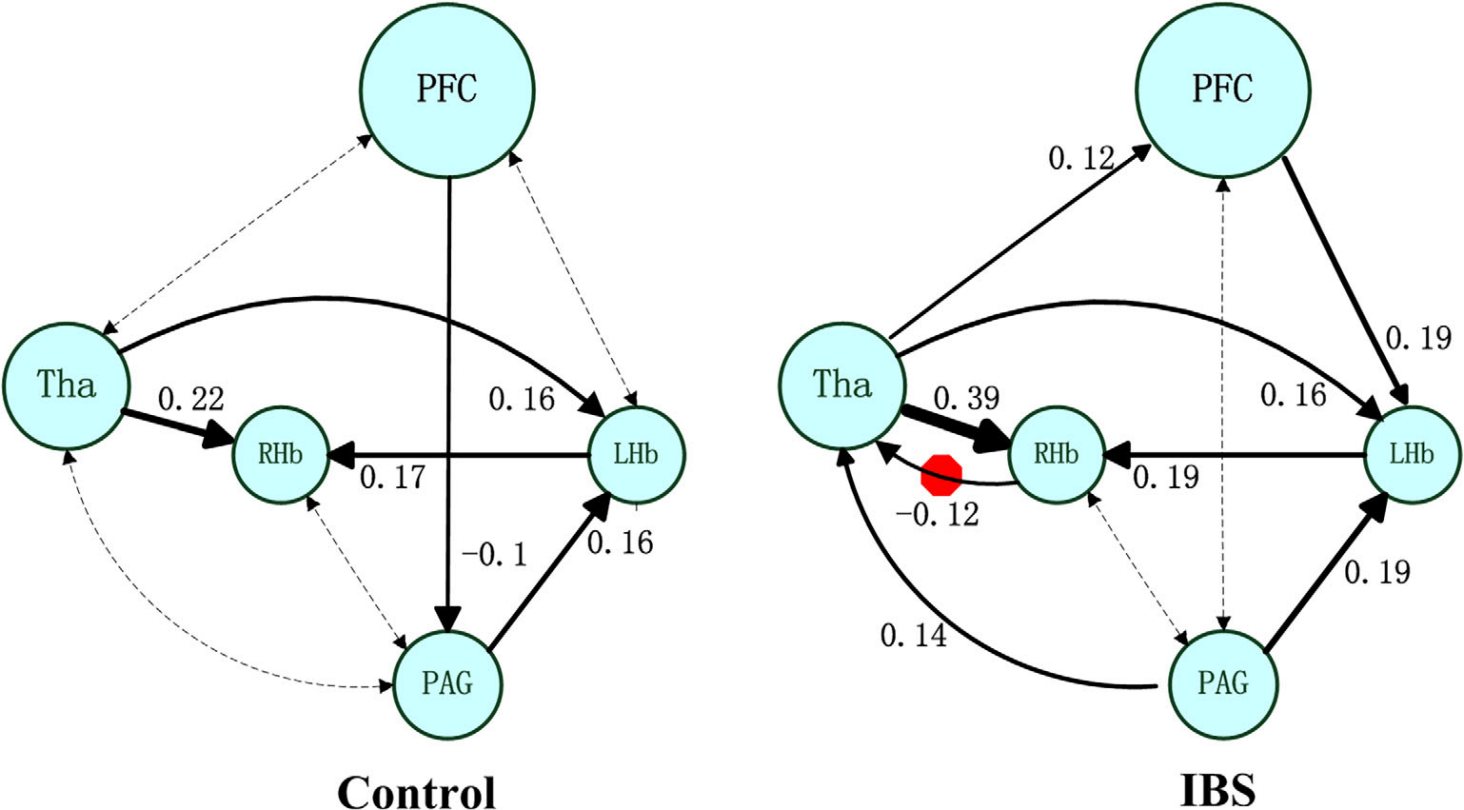
The winning model at the group level. The number shows the connectivity parameters (Hz) of the winning model in the IBS and healthy control groups. The solid lines represent connectivity values greater than 0.1 Hz, and their thickness shows the size of the value. The dotted lines represent the connectivity values below 0.1 Hz. The red octagon represents the significant group difference between healthy controls and patients with irritable bowel syndrome. IBS, irritable bowel syndrome; PFC, prefrontal cortex; Tha, thalamus; LHb, left habenula; RHb, right habenula; PAG, periaqueductal grey

All seven models analyzed in this study are shown in Figure 3a. At the group level, the fully connected model was found to be the best model in both the IBS and healthy control groups (Figure 3b). This model was the best model for 32 out of 34 healthy controls and 33 out of 34 IBS patients. The effective connectivity values are shown in Table 2. In the single‐group analysis, there was significant effective connectivity from the right thalamus to the right habenula (*p* = .019), from the PAG to the left habenula (*p* = .043), and from the left habenula to the PFC/right habenula (*p* = .025, *p* = .013) in the healthy controls. The IBS patients showed significant effective connectivity from the right thalamus to the PFC (*p* = .037), from the PAG to the right thalamus (*p* = .017), and from the left habenula to the right habenula (*p* < .001). In addition, DCM revealed significant bidirectional connections between the right thalamus and right habenula only in the IBS group (from the thalamus to habenula: *p* = .002; from the right habenula to thalamus: *p* = .003). The between‐group comparisons revealed that the IBS patients had significantly decreased effective connectivity from the right habenula to the right thalamus (*p* = .008). Finally, we reported effective connectivity only if their strength exceeded 0.1 Hz and their probability was greater than 0.95, and we discarded trivial connections. The winning model is shown in Figure 4.

**TABLE 2:** Mean connection strengths (in Hz) in the IBS and healthy control groups

Group	BMS	From PFC	From Tha	From LHb	From RHb	From PAG
Control	To PFC	0	0.055 ± 0.008	−0.097 ± 0.006*	0.041 ± 0.006	0.02 ± 0.007
	To Tha	−0.04 ± 0.008	0	−0.057 ± 0.006	0.044 ± 0.005	0.066 ± 0.007
	To LHb	−0.045 ± 0.009	0.157 ± 0.009	0	0.075 ± 0.006	0.162 ± 0.008*
	To RHb	−0.038 ± 0.009	0.215 ± 0.008*	0.166 ± 0.007*	0	0.023 ± 0.008
	To PAG	−0.101 ± 0.008	0.029 ± 0.008	−0.004 ± 0.006	−0.023 ± 0.006	0
IBS	To PFC	0	0.123 ± 0.009*	0.024 ± 0.006	−0.013 ± 0.006	−0.001 ± 0.008
	To Tha	−0.014 ± 0.009	0	0.06 ± 0.006	−0.119 ± 0.006**Δ	0.139 ± 0.008*
	To LHb	0.185 ± 0.01	0.156 ± 0.009	0	−0.01 ± 0.006	0.193 ± 0.009
	To RHb	−0.056 ± 0.01	0.385 ± 0.01**	0.191 ± 0.007**	0	0.015 ± 0.009
	To PAG	−0.037 ± 0.009	0.061 ± 0.009	−0.076 ± 0.006	−0.024 ± 0.006	0

*Note:* There are source regions in rows and target regions in columns.

Abbreviations: BMS, Bayesian Model Selection; IBS, irritable bowel syndrome; LHb, left habenula; PAG: periaqueductal grey; PFC: dorsolateral prefrontal cortex; RHb: right habenula; Tha, thalamus.

* *p* < .05.

** *p* < .01. The * represents the parameters that have significantly nonzero values revealed from single group *t* tests. Δ represents a significant between‐group difference in the habenular effective connectivity.

### 3.4 Habenular rsFC and effective connectivity in an independent cohort of IBS patients and healthy controls

The demographic data of the independent cohort are shown in Table S2. Consistent results were revealed in the independent cohort of IBS patients and healthy controls (Table S3). The patients with IBS showed enhanced rsFC of the habenula‐left dlPFC and habenula‐PAG, as well as decreased rsFC of the habenula‐left thalamus. In the DCM analysis, the fully connected model was found to be the best model in both the IBS and healthy control groups. This model was the best model for 19 out of 22 healthy controls and 19 out of 22 IBS patients. The mean connectivity parameters can be seen in Table S4. Group comparisons revealed that IBS had significantly decreased effective connectivity from the PAG to the right habenula (*p* = .037).

### 3.5 Classification performance by machine learning

IBS patients were classified with accuracies of 64.9%, 61.9%, 59.9% with SVM based on rsFC of the habenula‐dlPFC, habenula‐thalamus, and habenula‐PAG, respectively; this level of performance was further validated in the independent cohort of subjects (*N* = 44; accuracy = 62.7%, 55.5%, 57.0%, respectively). IBS patients were classified by combining three features (habenula‐dlPFC, habenula‐thalamus, and habenula‐PAG) with accuracies of 71.5% in the training cohort and 65.2% in the testing cohort.

In addition, we have done the permutation tests for the accuracy in the testing set, and the average *p*‐values are shown in Table S5. From the table, we can find that the model trained with all the three features has the lowest average *p*‐value of .026, which shows that the two classes can be well distinguished perfectly by combing these three features. Furthermore, the detailed results of the permutation test are listed in Table S6.

**FIGURE 5:**
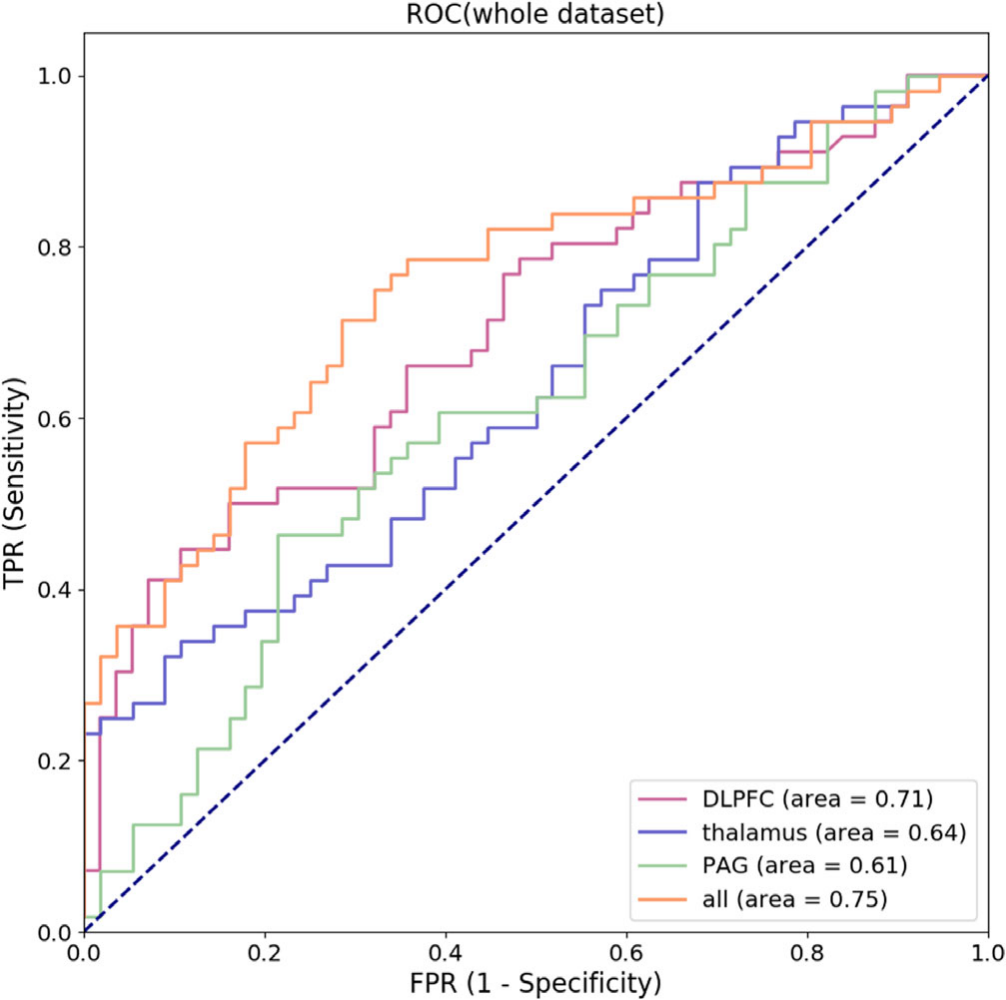
The receiver operating characteristic (ROC) curves. ROC, receiver operating characteristic; TPR, true positive rate; FPR, false positive rate; DLPFC, dorsolateral prefrontal cortex; PAG, periaqueductal grey

**FIGURE 6:**
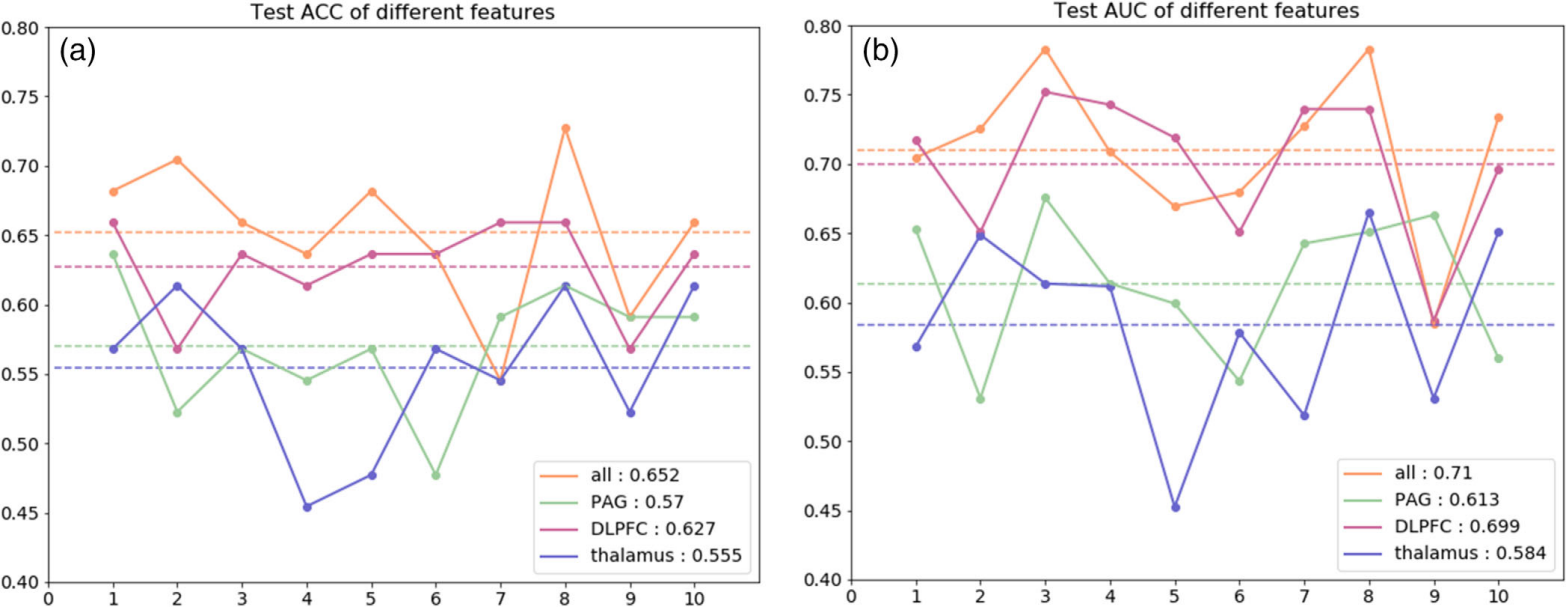
The model performance in different experiments. (a) The accuracy (ACC) values in the testing set for different features. The horizontal axis represents 10 splits, the vertical axis represents the values of ACC. The average ACC of an experiment is marked with dotted lines of the same color as broken line and listed in the lower right corner. (b) The area under curves (AUC) in testing set for different features. The description is the same as (a). ACC, accuracy; AUC, area under curve; DLPFC, dorsolateral prefrontal cortex; PAG, periaqueductal grey

Also, we have visualized the receiver operating characteristic (ROC) of the whole dataset in Figure 5. The areas under the ROC are 0.71, 0.64, and 0.61 for the three single features (rsFC of the habenula‐dlPFC, habenula‐thalamus, and habenula‐PAG), and the combination of all the three features has the largest AUC of 0.75. Furthermore, the ACC and AUC of the testing set are shown in Figure 6.

### 3.6 Correlation analysis

Habenula‐thalamus rsFC values were positively correlated with VAS scores (*r* = .467, *p* = .005, FDR corrected). There were no significant correlations between rsFC of the habenula‐DLPFC and VAS scores (*r* = .056, *p* = .754, FDR corrected) or disease duration (*r* = −.158, *p* = .373, FDR corrected) in the subjects with IBS. In addition, no significant correlations were suggested between rsFC of the habenula‐PAG and VAS scores (*r* = −.122, *p* = .493, FDR corrected) or disease duration (*r* = .073, *p* = .683, FDR corrected) in the IBS group. The effective connectivity from the right habenula to the right thalamus in IBS patients was negatively correlated with disease duration (*r* = −.407, *p* = .017, FDR corrected).

## 4 DISCUSSION

In this study, we characterized rsFC and effective connectivity of the habenula and the classification performance of machine learning in discriminating IBS patients from healthy controls. The results suggested different connectivity patterns of the habenula associated with pain characteristics in the subjects with IBS and healthy controls. Machine learning can help to classify IBS patients and healthy controls.

The habenula is composed of two subdivisions, the medial and the lateral habenula, in both rodents and humans (Boulos, Darcq, & Kieffer, 2017). This small structure receives inputs from the septal nuclei, limbic system, and basal ganglia (Namboodiri et al., 2016). Previous extensive animal research on the habenula has revealed a large spectrum of habenula‐related functions across emotional and cognitive brain processes (Boulos et al., 2017). In addition, the habenula has been identified as a brain structure contributing to mood disorders (Browne, Hammack, & Lucki, 2018; Lawson et al., 2017; Schafer et al., 2018) and acute/chronic pain (Erpelding et al., 2014; Shelton, Pendse, et al., 2012) in human research. Until now, no study had been carried out to explore the habenular mechanisms in IBS. We first explored habenular connectivity in subjects with IBS and demonstrated altered habenular rsFC and effective connectivity in this disease condition.

Different patterns of habenular rsFC were found in the IBS patients and healthy controls. First, enhanced rsFC of the habenula‐dlPFC and habenula‐PAG pathways were found in the subjects with IBS compared with the healthy controls. As a crossroad between the basal ganglia and the limbic system (Hikosaka, Sesack, Lecourtier, & Shepard, 2008), direct habenula‐prefrontal projections related to affective and cognitive processing have been identified by probabilistic tractography in humans (Vadovicova, 2014). Reduced rsFC between the habenula and the dlPFC, anterior mid‐cingulate cortex, and motor cortex have been reported in pediatric patients with complex regional pain syndrome (CRPS) (Erpelding et al., 2014). Lower rsFC of the left habenula‐ventrolateral PFC (vlPFC) and higher global efficiency of the habenula‐dlPFC in a reactive aggressive group (Gan et al., 2018) and the involvement of infralimbic‐amygdala‐habenula‐PAG interactions in fear conditioning (Barrett & Gonzalez‐Lima, 2018), together with the greater rsFC of the habenula‐PFC in subjects with higher depression levels (Ely et al., 2016), demonstrated the involvement of the habenula‐PFC pathway in emotion processing. In addition, the PAG is a crucial site in ascending and descending pain modulation (Lei, Sun, Lumb, & You, 2014; Samineni et al., 2017). IBS is often accompanied by chronic recurrent abdominal pain. According to the connectivity‐based segmentation of the PAG proposed by Ezra, Faull, Jbabdi, and Pattinson (2015), the PAG area in this study corresponds to the dorsomedial PAG, which has connectivity with the insular cortex, which is crucial in the perception, modulation and chronification of pain (Lu et al., 2016). Therefore, the altered rsFC of the habenula‐dlPFC and habenula‐PAG in subjects with IBS might indicate abnormal emotion and pain processing via the habenula in such a disease condition.

Moreover, decreased rsFC of the habenula‐thalamus pathway was also suggested in the subjects with IBS compared to the healthy controls, and these values were positively associated with pain intensity. The thalamus is a heterogeneous structure with differential functions across its subdivisions (Krauth et al., 2010). The connectivity difference in this study was located in the dorsolateral part of the thalamus, which has connectivity to the posterior parietal cortex (a probability of 75%) (Behrens et al., 2003) that is involved in integrating visual somatosensory and motor inputs (Dooley, Franca, Seelke, Cooke, & Krubitzer, 2014). Positive connectivity of the habenula‐thalamus in healthy subjects has been repeatedly reported in resting‐state fMRI studies (Ely et al., 2016; Ely, Stern, Kim, Gabbay, & Xu, 2019; Torrisi et al., 2017), and thalamo‐habenula projections are thought to be potentially involved in emotion modulation (Jesuthasan, 2018). The differences in the habenula‐thalamus rsFC associated with pain intensity in the IBS patients might indicate a dysregulation of emotion processing in such a chronic pain condition.

DCM revealed significantly decreased effective connectivity from the right habenula to the right thalamus in the IBS group compared with healthy control group, which was negatively associated with disease duration. The thalamo‐habenula projection was first evidenced in various vertebrates (Jesuthasan, 2018). Although resting‐state connectivity has been evidenced by many studies (Ely et al., 2016; Ely et al., 2019; Gan et al., 2018; Torrisi et al., 2017), the effective connectivity between the habenula and thalamus has scarcely been investigated. The significant effective connectivity from the right thalamus to the habenula in IBS patients indicated that the habenula receives information “driven” by the thalamus. Although the underlying mechanisms remain obscure, our study extends the physiological and behavioral implications of the habenula in IBS by demonstrating altered habenular effective connectivity in these disease conditions.

Machine learning was used in this study to classify IBS patients and healthy controls. Previous classification of pain conditions has depended mostly on self‐reports, which are less objective. Machine learning can train specific classifiers to best identify brain signatures in disease conditions. Attempts to distinguish different chronic pain conditions have been made in patients with chronic low back pain (Zhang et al., 2019). The classification accuracies in this study are similar to those reported by Zhang et al. (2019). Although the classification accuracy is not very high, the use of habenular rsFC in classifying IBS patients and healthy controls by an SVM classifier in this study represents an advance in our understanding of the brain's role in IBS and the ability to objectively classify individuals with the disease. Clinically, the pathology of IBS is heterogeneous, and multiple subtypes of IBS exist, including diarrhoea, constipation, or mixed (Chey, Kurlander, & Eswaran, 2015). Determining the predominant symptom plays an important role in the selection of diagnostic tests and treatments. IBS is sometimes not easily differentiated from other organic gastrointestinal diseases (Kim, Lin, & Pimentel, 2017). Further study of discriminating IBS subtypes and/or IBS from non‐IBS individuals by SVM may be more promising in the future.

The rsFC and effective connectivity patterns of the habenula in the independent cohort were largely consistent with the first cohort. Both cohorts showed altered functional connectivity and effective connectivity of the habenula in IBS patients compared to healthy controls. The discrepancy was in the finding that decreased effective connectivity existed in the habenula‐right thalamus in the first cohort, while it was in the habenula‐left thalamus in the second cohort. The effective connectivity changes were different in these two cohorts. The change was from the right thalamus to the habenula in the first cohort, while the change was from the PAG to the right habenula in the second cohort. Both showed decreased effective connectivity of the habenula. We did not differentiate IBS subtypes, which might have caused some heterogeneous results and need further study in the future.

One limitation that needs to be first considered in this study is that the habenula is a small structure, and defining its border is difficult. The seeds of the habenula used in the rsFC analysis were defined based on a previous report. This method in combination with manually tracing the habenula in high‐resolution T1‐weighted structural images (Lawson, Drevets, & Roiser, 2013) would provide greater reliability in future studies. Second, the between‐group differences in the rsFC values for the habenula‐thalamus and habenula‐PAG connections were not significant in the whole‐brain analysis. We used a smaller smoothing kernel and small‐volume correction and found connectivity alterations in these two pathways in the IBS group. This suggests the importance of smoothing kernel size and small‐volume correction in the evaluation of small ROIs. However, these results should be carefully interpreted. Moreover, the effect of medications on the results needed to be considered. We excluded patients taking opioids or NSAIDs and required all patients not to take medications during the 12 hr before the MR scan, which might have excluded the effects of drugs to some extent. Finally, IBS is often associated with psychological disorders such as anxiety and depression (Fond et al., 2014; Zamani, Alizadeh‐Tabari, & Zamani, 2019). The IBS sample in our study had almost no severe depressive symptoms, which limits the generality of the results. Future studies targeting IBS patients with depression are necessary to explore the effect of psychological comorbidities on IBS.

## 5 CONCLUSION

In summary, our results showed that IBS had altered resting‐state functional connectivity and effective connectivity of the habenula associated with pain, which might indicate dysregulation of affective and pain processing in these disease conditions. Machine learning can be a reliable classifier in differentiating IBS patients and healthy controls based on habenular rsFC. Our findings add to the growing body of neuroimaging evidence of the mechanisms underlying the pathophysiology of IBS.

## CONFLICT OF INTEREST

The authors declare no conflicts of interest.

## DATA AVAILABILITY STATEMENT

All the dataset are in house dataset and are available from the corresponding author upon reasonable request.

## Supporting information


**Appendix** **S1.** Supporting information
