# Supplementary material for: Altered resting‐state functional connectivity and effective connectivity of the habenula in irritable bowel syndrome: A cross‐sectional and machine learning study
Source: Hum Brain Mapp. 2020 Jun 3;41(13):3655–66. doi: 10.1002/hbm.25038 (PMC7416021; doi:10.1002/hbm.25038)
Supplement: Supplementary file 1 — Appendix S1. Supporting information [file HBM-41-3655-s001.doc]

**Supplemental materials**

**Medications**

The medications for all patients with irritable bowel syndrome (IBS) were recorded. Twelve patients were untreated, and ten patients choosed to modify their lifestyle (regular exercise, and/or dietary changes). Five patients took oral antispasmodics (Nifedipine, Domperidone). Three patients were treated with Chinese herbal medicine, and four patient were treated with Xiaoyao pill.

**Supplemental Table 1 Demographical and clinical data**

| Item | Group | | *P* |
| --- | --- | --- | --- |
|  | Control | IBS |  |
| Number of all subjects | 34 | 34 |  |
| Number of females (%) | 17(50%) | 17 (50%) | 0.596 |
| Age (years) | 44.9 ± 13.2^a^ | 44.9 ± 14.3 | 0.986 |
| Age range (years) | 24 – 63 | 22 – 68 |  |
| HDRS score | 1.2 ± 1.6 | 2.4 ± 3.6 | 0.09 |
| VAS score |  | 3.7 ± 2.0 |  |
| SF-MPQ score |  | 8.4 ± 5.1 |  |
| Disease duration (years) |  | 6.0 ± 7.3 |  |

Abbreviations: HDRS, Hamilton Depression Rating Scale; VAS, visual analogue scale; SF-MPQ, short-form McGill pain questionnaire; IBS, irritable bowel syndrome.

^a^ mean ± standard deviation; the unit of volume is cubic centimeters.

**Supplemental Table 2 Demographic data of the independent sample.**

| Item | Group | | *P* |
| --- | --- | --- | --- |
|  | Control | IBS |  |
| Number of subjects | 22 | 22 |  |
| Number of females (%) | 10(45%) | 10 (45%) | 0.62 |
| Age (years) | 36.3 ± 11.9 ^a^ | 35.2 ± 9.5 | 0.749 |
| Age range (years) | 22 – 60 | 23 – 62 | NA |
| HDRS score | 1.1 ± 0.9 | 2.5 ± 3.0 | 0.06 |
| VAS score | NA | 3.2 ± 2.1 | NA |
| SF-MPQ score | NA | 9.1 ± 6.8 | NA |
| Disease duration (years) | NA | 7.3 ± 6 | NA |

**Supplemental Table 3 Group differences of the habenula functional connectivity in the independent cohort.**

| Group difference | Target area | Volume data | | | Voxels | Z value |
| --- | --- | --- | --- | --- | --- | --- |
|  |  | X | Y | Z |  |  |
| Control < IBS | Left DLPFC | -46 | 22 | 28 | 461 | 3.5 |
|  | PAG | 6 | -34 | -8 | 25 | 3.2 |
| Control > IBS | Left thalamus | -22 | -24 | 8 | 11 | 2.6 |

IBS, irritable bowel syndrome; DLPFC, dorsolateral prefrontal cortex; PAG, periaqueductal gray.

**Supplemental Table 4 Mean connection strengths (in Hz) of the independent cohort of IBS and healthy controls**

| Group | BMS | From PFC | From Tha | From LHb | From RHb | From PAG |
| --- | --- | --- | --- | --- | --- | --- |
| Control | To PFC | 0 | 0.118±0.012 | -0.081±0.009 | -0.019±0.009 | 0.122±0.009 |
|  | To Tha | 0.048±0.009 | 0 | -0.012±0.009 | 0.004±0.009 | 0.089±0.01 |
|  | To LHb | 0.003±0.01 | 0.217±0.013 | 0 | 0.088±0.009 | 0.079±0.01 |
|  | To RHb | 0.168±0.01* | 0.156±0.013 | 0.215±0.009** | 0 | 0.184±0.01 |
|  | To PAG | 0.032±0.009 | -0.063±0.012 | 0.009±0.009 | -0.064±0.009 | 0 |
| IBS | To PFC | 0 | -0.103±0.012 | 0.009±0.008 | -0.051±0.007 | 0.059±0.011 |
|  | To Tha | -0.01±0.007 | 0 | 0.002±0.008 | -0.023±0.007 | 0.025±0.01 |
|  | To LHb | -0.13±0.01 | 0.004±0.013 | 0 | 0.154±0.008* | 0.074±0.012 |
|  | To RHb | -0.115±0.009 | 0.168±0.014 | 0.204±0.009* | 0 | -0.097±0.011^△^ |
|  | To PAG | -0.056±0.009 | -0.237±0.013 | 0.068±0.008 | 0.008±0.007 | 0 |

There are source regions in rows and target regions in columns. IBS, irritable bowel syndrome; BMS, Bayesian Model Selection; PFC: dorsolateral prefrontal cortex; Tha, thalamus; LHb, left habenula; RHb: right habenula; PAG: periaqueductal gray. *: *P* < 0.05; **, *P* < 0.01. The “*” represents the parameters that have significantly nonzero values revealed from single group *t*-tests. △, represents significant between-group difference of the habenula effective connectivity.

**Supplemental Table 5 The performance of each classification tasks**

| **Feature** | **Cross score** | **Training set** | | | | **Testing set** | | | | |
| --- | --- | --- | --- | --- | --- | --- | --- | --- | --- | --- |
|  |  | **ACC** | **AUC** | **Spe** | **Sen** | **ACC** | **p-value** | **AUC** | **Spe** | **Sen** |
| **Habenula-DLPFC** | 0.660 | 0.649 | 0.708 | 0.671 | 0.626 | 0.627 | 0.030 | 0.699 | 0.650 | 0.605 |
| **Habenula-thalamus** | 0.622 | 0.619 | 0.659 | 0.806 | 0.438 | 0.555 | 0.207 | 0.584 | 0.723 | 0.386 |
| **Habenula-PAG** | 0.582 | 0.599 | 0.610 | 0.529 | 0.668 | 0.570 | 0.121 | 0.613 | 0.518 | 0.623 |
| **all-features** | **0.694** | **0.715** | **0.776** | **0.750** | **0.679** | **0.652** | **0.026** | **0.710** | **0.732** | 0.573 |

DLPFC, dorsolateral prefrontal cortex; PAG, periaqueductal gray; ACC, accuracy; AUC, area under curve; Spe, specificity; Sen, sensitivity. p-value, the result of ACC in testing set accessed by permutation test.

**Supplemental Table 6 The classification indexes and parameters of the 10 splits.**

**Four classification tasks were done (Habenula-DLPFC, Habenula-thalamus, Habenula-PAG and all features), and each task has its own experimental results in 10 splits, respectively. Therefore, for each experiment, a sub table is given.**

1. **The detailed results of classification with the feature of Habenula-DLPFC.**

| **Habenula-DLPFC** | **Cross score** | **Training set** | | | | **Testing set** | | | | | ***C*** | ***gamma*** |  |
| --- | --- | --- | --- | --- | --- | --- | --- | --- | --- | --- | --- | --- | --- |
|  |  | **ACC** | **AUC** | **Spe** | **Sen** | **ACC** | **p-value** | **AUC** | **Spe** | **Sen** |  |  |  |
| ***set_1*** | 0.618 | 0.603 | 0.692 | 0.588 | 0.618 | 0.659 | 0.005 | 0.717 | 0.727 | 0.591 | 0.3 | 3 |  |
| ***set_2*** | 0.706 | 0.691 | 0.743 | 0.706 | 0.676 | 0.568 | 0.040 | 0.651 | 0.545 | 0.591 | 0.4 | 1 |  |
| ***set_3*** | 0.676 | 0.647 | 0.679 | 0.676 | 0.618 | 0.636 | 0.000 | 0.752 | 0.591 | 0.682 | 5 | 0.3 |  |
| ***set_4*** | 0.647 | 0.676 | 0.701 | 0.588 | 0.765 | 0.614 | 0.008 | 0.743 | 0.727 | 0.500 | 0.3 | 0.3 |  |
| ***set_5*** | 0.662 | 0.618 | 0.705 | 0.588 | 0.647 | 0.636 | 0.011 | 0.719 | 0.727 | 0.545 | 0.3 | 1 |  |
| ***set_6*** | 0.662 | 0.662 | 0.753 | 0.647 | 0.676 | 0.636 | 0.038 | 0.651 | 0.636 | 0.636 | 0.3 | 2 |  |
| ***set_7*** | 0.632 | 0.574 | 0.676 | 0.706 | 0.441 | 0.659 | 0.002 | 0.740 | 0.636 | 0.682 | 2 | 0.6 |  |
| ***set_8*** | 0.647 | 0.676 | 0.643 | 0.912 | 0.441 | 0.659 | 0.004 | 0.740 | 0.864 | 0.455 | 9 | 8 |  |
| ***set_9*** | 0.706 | 0.706 | 0.768 | 0.706 | 0.706 | 0.568 | 0.177 | 0.587 | 0.545 | 0.591 | 0.5 | 5 |  |
| ***set_10*** | 0.647 | 0.632 | 0.715 | 0.588 | 0.676 | 0.636 | 0.012 | 0.696 | 0.500 | 0.773 | 0.8 | 0.5 |  |
| **avg** | **0.660** | **0.649** | **0.708** | **0.671** | **0.626** | **0.627** | **0.030** | **0.699** | **0.650** | **0.605** |  |  |  |

**(b) The detailed results of classification with the feature of Habenula-thalamus.**

| **Habenula-thalamus** | **Cross score** | **Training set** | | | | **Testing set** | | | | | ***C*** | ***gamma*** |
| --- | --- | --- | --- | --- | --- | --- | --- | --- | --- | --- | --- | --- |
|  |  | **ACC** | **AUC** | **Spe** | **Sen** | **ACC** | **p-value** | **AUC** | **Spe** | **Sen** |  |  |
| ***set_1*** | 0.574 | 0.559 | 0.683 | 0.618 | 0.500 | 0.568 | 0.206 | 0.568 | 0.682 | 0.455 | 1 | 1 |
| ***set_2*** | 0.588 | 0.603 | 0.637 | 0.912 | 0.294 | 0.614 | 0.038 | 0.649 | 0.864 | 0.364 | 0.3 | 8 |
| ***set_3*** | 0.647 | 0.647 | 0.633 | 0.912 | 0.382 | 0.568 | 0.081 | 0.614 | 0.909 | 0.227 | 0.5 | 6 |
| ***set_4*** | 0.647 | 0.632 | 0.673 | 0.647 | 0.618 | 0.455 | 0.087 | 0.612 | 0.500 | 0.409 | 3 | 0.3 |
| ***set_5*** | 0.721 | 0.706 | 0.795 | 0.794 | 0.618 | 0.477 | 0.698 | 0.452 | 0.273 | 0.682 | 5 | 9 |
| ***set_6*** | 0.618 | 0.647 | 0.626 | 0.941 | 0.353 | 0.568 | 0.159 | 0.579 | 0.818 | 0.318 | 2 | 2 |
| ***set_7*** | 0.662 | 0.647 | 0.653 | 0.912 | 0.382 | 0.545 | 0.394 | 0.519 | 0.864 | 0.227 | 0.4 | 9 |
| ***set_8*** | 0.559 | 0.559 | 0.619 | 0.824 | 0.353 | 0.614 | 0.030 | 0.665 | 0.864 | 0.364 | 0.3 | 7 |
| ***set_9*** | 0.588 | 0.588 | 0.695 | 0.588 | 0.588 | 0.523 | 0.336 | 0.531 | 0.591 | 0.455 | 0.6 | 0.8 |
| ***set_10*** | 0.618 | 0.603 | 0.572 | 0.912 | 0.294 | 0.614 | 0.038 | 0.651 | 0.864 | 0.364 | 3 | 2 |
| **avg** | **0.622** | **0.619** | **0.659** | **0.806** | **0.438** | **0.555** | **0.207** | **0.584** | **0.723** | **0.386** |  |  |

**(c) The detailed results of classification with the feature of Habenula-PAG.**

| **Habenula-PAG** | **Cross score** | **Training set** | | | | **Testing set** | | | | | ***C*** | ***gamma*** |
| --- | --- | --- | --- | --- | --- | --- | --- | --- | --- | --- | --- | --- |
|  |  | **ACC** | **AUC** | **Spe** | **Sen** | **ACC** | **p-value** | **AUC** | **Spe** | **Sen** |  |  |
| ***set_1*** | 0.559 | 0.603 | 0.556 | 0.735 | 0.471 | 0.636 | 0.034 | 0.653 | 0.818 | 0.455 | 0.3 | 8 |
| ***set_2*** | 0.618 | 0.662 | 0.633 | 0.676 | 0.647 | 0.523 | 0.335 | 0.531 | 0.591 | 0.455 | 4 | 10 |
| ***set_3*** | 0.559 | 0.574 | 0.580 | 0.294 | 0.853 | 0.568 | 0.022 | 0.676 | 0.227 | 0.909 | 1 | 0.4 |
| ***set_4*** | 0.588 | 0.632 | 0.618 | 0.618 | 0.647 | 0.545 | 0.086 | 0.614 | 0.545 | 0.545 | 0.3 | 5 |
| ***set_5*** | 0.574 | 0.632 | 0.614 | 0.676 | 0.588 | 0.568 | 0.103 | 0.599 | 0.727 | 0.409 | 4 | 9 |
| ***set_6*** | 0.632 | 0.618 | 0.676 | 0.500 | 0.735 | 0.477 | 0.295 | 0.543 | 0.318 | 0.636 | 0.8 | 0.6 |
| ***set_7*** | 0.559 | 0.544 | 0.589 | 0.235 | 0.853 | 0.591 | 0.044 | 0.643 | 0.318 | 0.864 | 0.3 | 0.3 |
| ***set_8*** | 0.544 | 0.529 | 0.592 | 0.324 | 0.735 | 0.614 | 0.041 | 0.651 | 0.500 | 0.727 | 3 | 0.5 |
| ***set_9*** | 0.574 | 0.588 | 0.586 | 0.529 | 0.647 | 0.591 | 0.021 | 0.663 | 0.636 | 0.545 | 0.3 | 0.3 |
| ***set_10*** | 0.618 | 0.603 | 0.651 | 0.706 | 0.500 | 0.591 | 0.232 | 0.560 | 0.500 | 0.682 | 5 | 10 |
| **avg** | **0.582** | **0.599** | **0.610** | **0.529** | **0.668** | **0.570** | **0.121** | **0.613** | **0.518** | **0.623** |  |  |

**(d) The detailed results of classification with all three features.**

| **all-features** | **Cross score** | **Training set** | | | | **Testing set** | | | | | ***C*** | ***gamma*** |
| --- | --- | --- | --- | --- | --- | --- | --- | --- | --- | --- | --- | --- |
|  |  | **ACC** | **AUC** | **Spe** | **Sen** | **ACC** | **p-value** | **AUC** | **Spe** | **Sen** |  |  |
| ***set_1*** | 0.662 | 0.662 | 0.743 | 0.794 | 0.529 | 0.682 | 0.012 | 0.705 | 0.909 | 0.455 | 0.3 | 0.3 |
| ***set_2*** | 0.706 | 0.706 | 0.794 | 0.647 | 0.765 | 0.705 | 0.002 | 0.725 | 0.727 | 0.682 | 5 | 0.9 |
| ***set_3*** | 0.662 | 0.662 | 0.717 | 0.735 | 0.588 | 0.659 | 0.000 | 0.783 | 0.818 | 0.500 | 0.6 | 0.3 |
| ***set_4*** | 0.706 | 0.779 | 0.791 | 0.853 | 0.706 | 0.636 | 0.011 | 0.709 | 0.818 | 0.455 | 7 | 1 |
| ***set_5*** | 0.662 | 0.735 | 0.834 | 0.735 | 0.735 | 0.682 | 0.026 | 0.669 | 0.773 | 0.591 | 1 | 5 |
| ***set_6*** | 0.735 | 0.750 | 0.802 | 0.765 | 0.735 | 0.636 | 0.022 | 0.680 | 0.636 | 0.636 | 0.5 | 0.3 |
| ***set_7*** | 0.691 | 0.691 | 0.719 | 0.912 | 0.471 | 0.545 | 0.003 | 0.727 | 0.727 | 0.364 | 0.3 | 2 |
| ***set_8*** | 0.676 | 0.676 | 0.716 | 0.647 | 0.706 | 0.727 | 0.001 | 0.783 | 0.727 | 0.727 | 0.3 | 0.3 |
| ***set_9*** | 0.721 | 0.735 | 0.816 | 0.647 | 0.824 | 0.591 | 0.176 | 0.585 | 0.636 | 0.545 | 0.3 | 0.3 |
| ***set_10*** | 0.721 | 0.750 | 0.824 | 0.765 | 0.735 | 0.659 | 0.005 | 0.733 | 0.545 | 0.773 | 0.8 | 8 |
| **avg** | **0.694** | **0.715** | **0.776** | **0.750** | **0.679** | **0.652** | **0.026** | **0.710** | **0.732** | **0.573** |  |  |

DLPFC, dorsolateral prefrontal cortex; PAG, periaqueductal gray; ACC, accuracy; AUC, area under curve; Spe, specificity; Sen, sensitivity.

p-value, the result of ACC in testing set accessed by permutation test.
